# *Salmonella* takes control: effector-driven manipulation of the host

**DOI:** 10.1016/j.mib.2008.12.001

**Published:** 2009-02

**Authors:** Emma J McGhie, Lyndsey C Brawn, Peter J Hume, Daniel Humphreys, Vassilis Koronakis

**Affiliations:** University of Cambridge, Department of Pathology, Tennis Court Road, Cambridge CB2 1QP, UK

## Abstract

*Salmonella* pathogenesis relies upon the delivery of over thirty specialised effector proteins into the host cell via two distinct type III secretion systems. These effectors act in concert to subvert the host cell cytoskeleton, signal transduction pathways, membrane trafficking and pro-inflammatory responses. This allows *Salmonella* to invade non-phagocytic epithelial cells, establish and maintain an intracellular replicative niche and, in some cases, disseminate to cause systemic disease. This review focuses on the actions of the effectors on their host cell targets during each stage of *Salmonella* infection.

## Introduction

**Figure 1:**
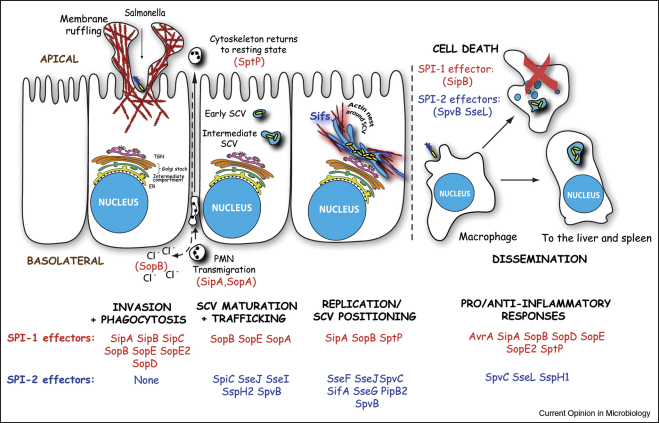
Schematic representation of the major stages underlying *Salmonella* infection. *Salmonellae* invade non-phagocytic cells by inducing membrane deformation and rearrangement of the underlying actin cytoskeleton (membrane ruffling), enclosing bacteria in intracellular phagosomal compartments termed *Salmonella*-containing vacuoles (SCVs). SCVs traffic towards the perinuclear region of the host cell and mature via selective interactions with the endocytic pathway. Once the SCV is positioned next to the Golgi apparatus, intracellular bacterial replication begins. This stage is characterised by the formation of SCV tubulovesicular structures called *Salmonella*-induced filaments (Sifs) and the accumulation of F-actin around the bacterial phagosome (actin nest). Chloride ion (Cl^-^) secretion and polymorphonuclear leukocyte (PMN) transmigration contribute towards diarrhoea and intestinal inflammation. In addition, *Salmonella* manipulates specific host immune response pathways. *Salmonella* serovars associated with systemic disease are able to enter intestinal macrophages, inducing cell death as well as using them as a vehicle to disseminate to the liver and spleen via the bloodstream and lymphatic system. SPI-1 and SPI-2 effectors involved in each individual infection stage are indicated. Note that SPI-1 and SPI-2 effectors do not operate sequentially and independently of one another as previously thought. Instead, both subsets play key roles in SCV maturation, positioning and replication (Abbreviations: ER, endoplasmic reticulum; TGN, trans-Golgi network).

*Salmonellae* are enteropathogenic Gram-negative bacteria that infect humans and animals, causing each year *∼*1.3 billion cases of human disease ranging from diarrhoea to systemic typhoid fever. Following ingestion by the host, *Salmonella* invades the intestinal mucosa via several routes. Bacteria may be taken up by antigen-sampling M cells, be captured in the lumen by CD18-expressing phagocytes that penetrate the epithelial monolayer, or may force their own entry into non-phagocytic enterocytes. Upon internalisation into non-phagocytic cells, *Salmonella* becomes enclosed within an intracellular phagosomal compartment termed the *Salmonella*-containing vacuole (SCV). The maturing SCV traffics towards the Golgi apparatus, undergoing selective interactions with the host endocytic pathway. Once positioned within the perinuclear area, the SCV-enclosed bacteria replicate, a stage characterised by formation of tubulovesicular SCV structures called *Salmonella*-induced filaments (Sifs). Although most *Salmonella* infections remain localised to the intestine, where stimulation of inflammatory responses contributes to diarrhoea, in typhoid disease *Salmonella* survives in intestinal macrophages, disseminating to the liver and spleen via the bloodstream and lymphatic system (Figure 1). This multi stage infection of the host is directed by *Salmonella*-mediated delivery of an array of specialised effector proteins into the eukaryotic host cells via two distinct type III secretion systems (T3SSs), encoded by pathogenicity islands 1 (SPI-1 T3SS) and 2 (SPI-2 T3SS). Additional secretion systems, including the *sci*-encoded (*Salmonella enterica c*entisome 7 genomic *i*sland) type VI secretion system [1] and the ZirTS pathway [2], appear to be functional during *Salmonella* infection and have been demonstrated to contribute towards virulence. However, these systems are not currently well characterised compared to the SPI-1 and SPI-2 T3SSs.

Over thirty SPI-1 and SPI-2 T3SS effectors have been shown to manipulate a succession of key host cellular functions, including signal transduction, membrane trafficking and pro-inflammatory immune responses (Tables 1 and 2, see Supplementary information for fully referenced versions). In this review, we will summarise the actions of these effectors on their host cell targets and indicate emerging examples of effector cooperation.

## Effector-mediated forced entry into non-phagocytic epithelial cells

A subset of delivered SPI-1 effectors (SipA, SipC, SopB, SopD, SopE, SopE2) function to induce membrane deformation and rearrangement of the underlying actin cytoskeleton (‘membrane ruffling’), triggering bacterial internalisation into SCVs.

The C-terminus of the SPI-1 T3SS translocon component SipC (SspC) directly nucleates actin assembly leading to rapid filament growth from barbed ends, whereas its N-terminus bundles actin filaments [3]. Although not necessary for *Salmonella* entry, SipA (SspA) increases invasion efficiency into cultured cells [3] and enhances *Salmonella* enterocolitis *in vivo* [4]. SipA promotes actin polymerisation by reducing the critical concentration for actin assembly [3] and binds to F-actin with high affinity, resulting in mechanical stabilisation of filaments [3,5,6]. SipA potentiates the actin nucleating and bundling activities of SipC [3] and enhances the activity of the host actin bundling protein T-plastin (fimbrin) [3,6]. SipA also prevents binding of the cellular actin depolymerising proteins ADF/cofilin to F-actin and displaces pre-bound ADF/cofilin from F-actin [5]. The F-actin severing activity of cellular gelsolin was originally reported to be prevented by SipA [5], but a later study showed that higher concentrations of gelsolin were able to partially sever SipA-F-actin complexes [7]. In addition, SipA is able to reanneal gelsolin-severed and -capped actin filament fragments [5,7].

In contrast to SipA and SipC, SopE and SopE2 do not bind actin. They modulate the host actin cytoskeleton indirectly by mimicking cellular guanine exchange factors (GEFs) [6]. In particular, they catalyse the exchange of bound GDP for GTP to activate host Rho GTPases that stimulate downstream pathways that drive actin cytoskeletal assembly via Arp2/3. *In vitro*, SopE and SopE2 have differing substrate specificities; while SopE activates Rac-1 and Cdc42, SopE2 appears to exhibit specificity for Cdc42 [6]. SopE-dependent activation of Rac-1 alone appears sufficient for bacterial internalisation [8^•^].

The inositol phosphatase SopB (SigD) dephosphorylates a range of phosphoinositide phosphate and inositol phosphate substrates *in vitro* [4,6]. Inhibition of SopB phosphoinositide phosphatase activity attenuates *Salmonella*-induced cytoskeletal reorganisation [6]. Recent work indicates that SopB-dependent stimulation of the cellular SH3-containing guanine nucleotide exchange factor, SGEF, activates the small GTPase RhoG, which contributes to the actin remodelling that occurs during *Salmonella* entry [8^•^].

SopB-dependent hydrolysis of PI(4,5)P_2_ at the ruffling host membrane enhances the subsequent annealing of plasmalemmal invaginations to rapidly enclose bacteria within a sealed phagosome (the SCV) [6]. In addition, SopB-dependent formation of PI(3)P at the host plasma membrane has been reported to contribute towards the formation of larger, more stable macropinosomes [6] and has been shown to facilitate bacterial phagocytosis by recruiting the host SNARE protein VAMP8 [9]. Another effector, SopD, cooperates with SopB to aid membrane fission and macropinosome formation [10].

Following engulfment *Salmonellae* return the host cell cytoskeleton back to its resting state, an event mediated by the N-terminal GTPase activation (GAP) domain of SptP. This stimulates the intrinsic GTPase activities of SopE/SopE2/SopB-activated Cdc42 and Rac-1, causing their downregulation [6].

## Maturation and trafficking of the *Salmonella*-containing vacuole

When SCVs form, they acquire transiently cellular markers associated with the early endocytic pathway, e.g*.* the transferrin receptor (TfnR), early endosomal antigen 1 (EEA1) and several Rab GTPases such as Rab4, Rab5 and Rab11, and SCVs mature in a Rab7-dependent manner [11]. SCVs may then uncouple from the endocytic pathway, to avoid lysosomal fusion [11], although recent evidence suggests that late endosome/lysosome (LE/Lys) content is continually delivered to the SCV in a Rab7- and microtubule-dependent manner [12^•^]. Regardless of whether this LE/Lys interaction occurs, as the SCV matures, early markers are sequentially replaced by late endosome/lysosome markers including Rab7, vacuolar ATPase (v-ATPase) and lysosomal membrane glycoproteins (lpgs) e.g*.* LAMP-1 [11].

SopE and the inositol phosphatase activity of SopB are required for SCV recruitment of Rab5 [13,14^••^], which binds the phosphatidylinositol 3-kinase Vps34 required for LAMP-1 recruitment [11,14^••^]. Vps34 in turn generates PI(3)P on the SCV membrane [14^••^], which is necessary for the recruitment of EEA1 [11]. SopB also inhibits the degradation of epidermal growth factor receptors (EGFR) by lysosomes [15], and has been recently shown to recruit sorting nexin-1 (SNX-1), which likely contributes to the disappearance of late endosomal/lysosomal markers, such as the mannose 6-phosphate receptor, from the maturing SCV [16]. These observations in combination suggest that SopB plays a key role in diverting SCV trafficking from the endosomal maturation pathway. SopB is additionally required for activation of Akt [4], which in turn deactivates the Rab14 GAP, AS160. Activated Rab14 increases intracellular *Salmonella* replication, possibly by delaying SCV-lysosomal fusion [17^••^]. In addition, SpiC is thought to prevent fusion of macrophage late endosomes/lysosomes with the SCV [11].

The SPI-2 effector SseJ is required for full virulence during systemic infection of mice and localises to SCVs [18]. It has deacylase activity *in vitro* [19] and during *Salmonella* infection it esterifies cholesterol, a lipid enriched in SCV membranes. SseJ also exhibits phospholipase A activity [20].

The SPI-1 effector SopA structurally and functionally mimics cellular HECT E3 ubiquitin ligases [21^•^], promoting bacterial escape from the SCV in HeLa cells. It may, therefore, have a role in disrupting SCV integrity [11] although the significance of this activity is unclear.

Several hours post-infection of host cells, an F-actin meshwork assembles around the replicative SCV [11,22], which appears to be bound and stabilised by SipA [23]. Several SPI-2 effectors may also regulate SCV-associated actin dynamics. In particular, the kinase SteC is essential for the formation of SCV-associated F-actin [22], while SseI and SspH2 co-localise with SCV-associated F-actin and bind the host actin-crosslinking protein filamin [24]. Furthermore, SspH2 interacts with the cellular G-actin binding protein profilin and inhibits actin polymerisation rates *in vitro* [24]. The plasmid-encoded effector SpvB ADP ribosylates monomeric actin preventing its polymerisation and inhibits the formation of SCV-associated F-actin [24].

## SCV positioning and formation of *Salmonella*-induced filaments (Sifs)

As it matures, the SCV migrates towards the perinuclear region of the host cell by transiently recruiting the Rab7-interacting lysosomal protein (RILP), which in turn associates with the minus end-directed microtubule motor, dynein [25]. Maintaining the SCV within the perinuclear region appears to be important for promoting bacterial replication. The close proximity of the SCV to the Golgi may facilitate interception of endocytic and exocytic transport vesicles to obtain nutrients and/or membrane [25]. In support of this, SifA, SseG and SseF are required for re-direction of exocytic transport vesicles to the SCV [26^•^].

SseG and SseF have been suggested to maintain the SCV in the perinuclear region by forming a functional complex [27] that either ‘tethers’ the SCV to the Golgi apparatus or manipulates dynein activity [25]. By contrast, SifA binds the host protein SKIP (SifA and kinesin interacting protein) to downregulate PipB2-induced recruitment of the plus end-directed microtubule motor kinesin to the SCV [28^•^,29^••^]. Efficient localisation of SifA to the SCV is mediated by the SPI-1 effector SipA [23]. SopB-mediated phosphorylation of the actin-associated motor myosin II light chain (MLC), most likely via the Rho/ROCK/MLC signalling pathway, is also required for retention of the SCV within the perinuclear region of the host cell [30^•^].

Once the SCV is positioned, the bacteria begin to replicate. This replicative stage is characterised by the formation of LAMP-rich specialised tubulovesicular structures termed *Salmonella*-induced filaments (Sifs) that extend away from the SCV along the microtubule network. Sifs are thought to be generated by fusion of late endosomes/lysosomes with the SCV [11], although their precise role in infection is undetermined. SifA is essential for Sif formation [11] and maintenance of SCV integrity [18]. Its transient overexpression is sufficient to induce swelling and aggregation of late endosomes and formation of Sif-like tubules in mammalian cells [11]. Although the molecular mechanism by which SifA induces Sif formation is unclear, the effector has been shown to interact with Rab7 and is suggested to promote Sif extension by uncoupling Rab7 from RILP, preventing the recruitment of dynein to Sifs [25]. The SPI-2 effector PipB2 also promotes Sif extension, most probably through a direct interaction with kinesin-1 [28^•^,31].

Both SseF and SseG are thought to augment Sif formation by modulating the aggregation of endosomal compartments. *Salmonella* mutants lacking *sseF, sseG* or another SPI-2 effector gene, s*opD2* induce fewer Sifs compared with wild type bacteria, but form a greater number of filamentous aggregates with punctate LAMP-1 distribution within infected cells. These ‘pseudo-Sifs’ may represent Sif precursors [25,32].

By contrast, both SseJ and SpvB antagonise Sif formation. Mutation of *sseJ* or *spvB* increases the number of Sifs [25], and transfection of epithelial cells with SseJ before *Salmonella* infection inhibits Sif formation [18]. SseJ activity also appears to be required for loss of SCV integrity as, in contrast to a *sifA* mutant, a *sifA sseJ* double mutant retains its vacuolar membrane [18].

## Modulation of the innate immune response and host cell death

SPI-1 effectors additionally induce acute intestinal inflammation, a hallmark of *Salmonella* infection. Stimulation of Cdc42 by SopE/SopE2/SopB during *Salmonella* invasion leads to Raf1-dependent upregulation of Erk, Jnk and p38 mitogen-activated protein kinase (MAPK) pathways and subsequent activation of the transcription factors AP-1 and NFκB [4,6,8^•^]. This results in the release of proinflammatory cytokines including IL-8, stimulating the recruitment of polymorphonuclear leukocytes (PMNs). Simultaneously, a SipA N-terminal region triggers a novel Arf6- and phospholipase D signalling cascade that activates protein kinase Cα, leading to apical secretion of the potent PMN chemoattractant hepoxillin A3 [4,33]. This promotes PMN transmigration across the epithelium into the intestinal lumen [4,33], which is probably augmented by the E3 ubiquitin ligase activity of SopA [21^•^]. PMN transmigration appears to contribute towards diarrhoeal disease, enhancing *Salmonella* transmission via the faecal-oral route. Ins(1,4,5,6)P_4_ production via SopB inositol phosphatase activity also plays a role in the induction of diarrhoea by promoting cellular chloride ion secretion and fluid flux [4,6], while SopD contributes towards enteritis in infected calves through an unknown mechanism [4]. Disruption of intestinal epithelial cell tight junctions by SopB, SopE, SopE2 and SipA is also likely to promote fluid flux and PMN transmigration [34], although intriguingly, another SPI-1 effector, AvrA has been recently shown to counteract this activity [35].

Inflammatory responses are further augmented by effector-induced macrophage cell death. This was thought to be due to direct activation of caspase-1 by SipB, resulting in release of proinflammatory cytokines [4], but has been shown to depend on the delivery of flagellin into the macrophage cytosol, possibly via the SPI-1 T3SS [36]. SipB additionally triggers a delayed caspase-1-independent cell death [4,36]. More recently, SpvB and SseL have been reported to induce a slower SPI-2-dependent cell death pathway [37,38^•^].

*Salmonella* also deliver effectors that suppress cellular immune responses. Both SptP GAP and tyrosine phosphatase activities play a role in reversing MAPK activation [39,40] and AvrA acetyltransferase activity towards specific mitogen-activated protein kinase kinases (MAPKKs) prevents Jnk activation [41^•^]. SpvC also directly inhibits Erk, Jnk and p38 MAPKs through its phosphothreonine lyase activity [42,43].

Finally, *Salmonella* targets transcription factors downstream of MAPK pathways. The SPI-2 deubiquitinase SseL suppresses NFκB activation by impairing IκBα ubiquitination and degradation [44], an activity also reported for AvrA [45]. SspH1 additionally inhibits NFκB-dependent gene expression, possibly via ubiquitination of the host cell kinase PKN1 [46].

## Perspectives–effector localisation and cooperation

*Salmonellae* have evolved an array of subversive SPI-1 and SPI-2 effector proteins with diverse biochemical activities. The actions of individual SPI-1 effectors during *Salmonella* entry have been intensively studied, but it is not clear which effectors are present in the host cell at any one time, nor are the sequence and kinetics of effector translocation established, although work has begun to dissect this complex process [47]. Likewise, it is still not completely understood how the discrete activities of all of these effectors are controlled, though effectors do appear to have varied half-lives following their translocation [6]. Work on the localisation of effectors has shown that in addition to the translocase SipB, six other SPI-1 effectors (SipA, SipC, SopB, SopE, SopE2 and SptP) are delivered to the host plasma membrane, suggesting that this may provide an interface for effector–effector interplay [48] as well as effector-target interaction(s) [49] during bacterial entry. Combinatorial screens [50] have confirmed known/proposed effector interactions [3,48], and suggested two novel cooperative associations, SipC–SopB and SipC–SopE [50], which require further investigation. However, interactions of SPI-2 effectors with their host cell targets, as well as with each other is a less well-understood area of *Salmonella* pathogenesis.

Recent work has challenged the conventional view that SPI-1 effectors solely mediate *Salmonella* invasion and SCV biogenesis, while SPI-2 effectors promote intracellular bacterial replication and systemic spread. SipA [23], SopB [30^•^] and SptP (Humphreys *et al*., unpublished) all persist in host cells hours after bacterial invasion and have key roles in SCV positioning and/or intracellular replication (Figure 1), suggesting possible interplay between SPI-1 and SPI-2 effectors. Indeed, SipA has already been shown to cooperate with SifA to mediate perinuclear SCV positioning [23]. Continued studies of effector action and interplay seem likely to explain further the processes underlying infection and highlight new facets of eukaryotic cell biology.

## References and recommended reading

Papers of particular interest have been highlighted as:

• of special interest
•• of outstanding interest

## Figures and Tables

**Table 1:** Effectors requiring the SPI-1-encoded T3SS for their translocation.

Effector	Gene location	Selected homologues	Activity	Host cell target(s)	Role in infection
AvrA	SPI-1	*Yersinia* YopJ/P	Cysteine protease with deubiquitinase activity, acetyltransferase	MKK4/7, IκBα, β-catenin	Inhibits inflammation, represses apoptosis & epithelial innate immunity
SipA (SspA)	SPI-1	*Shigella* IpaA	Actin binding/stabilising	Actin, T-plastin	Increases internalisation efficiency, enhances actin assembly, potentiates SipC activity, triggers PMN transmigration, maintains perinuclear SCV positioning, disrupts tight junctions
SipB (SspB)	SPI-1	*Shigella* IpaB	SPI-1 TTSS translocon component	Cholesterol	SPI-1 effector delivery, apoptosis of phagocytes
SipC (SspC)	SPI-1	*Shigella* IpaC	SPI-1 TTSS translocon component, actin nucleation & bundling	Actin	SPI-1 effector delivery, induces membrane ruffling
SipD (SspD)	SPI-1	*Shigella* IpaD			Regulates SPI-1 effector secretion
SopA	Outside SPI-1	Putative EHEC effector (Genbank NP_309587.1)	E3 ubiquitin ligase	HsRMA1	Disrupts SCV integrity, induces PMN transmigration
SopB (SigD)	SPI-5	*Shigella* spp. IpgD, cellular 4-phosphatases, synaptojanin	Inositol polyphosphate phosphatase	Inositol phosphates	Promotes membrane fission & macropinosome formation, maintains perinuclear SCV positioning, promotes epithelial cell survival, triggers nitric oxide production in macrophages, promotes fluid secretion, disrupts tight junctions
SopE	Bacteriophage SopEϕ	*Salmonella* SopE2	Guanine exchange factor (GEF) mimic	Rac-1, Cdc42	Induces membrane ruffling & proinflammatory responses, promotes fusion of SCV with early endosomes, disrupts tight junctions
SopE2	In vicinity of bacteriophage remnants	*Salmonella* SopE	GEF mimic	Cdc42	Induces membrane ruffling & proinflammatory responses, increases macrophage iNos expression, disrupts tight junctions
SptP	SPI-1	N-terminus: *Yersinia* YopE, *Pseudomonas aeruginosa* ExoS. C-terminus: cellular tyrosine phosphatases, *Yersinia* YopH	GTPase activating protein (GAP) mimic, tyrosine phosphatase	Cdc42, Rac-1, vimentin	Returns host cytoskeleton to resting state following bacterial entry, downregulates proinflammatory responses
SlrP^a^	Outside SPI-1/SPI-2	*Salmonella* SspH1/H2, GogB, *Yersinia* YopM, *Shigella* IpaH_7.8/9.8_	Ubiquitin ligase?		Confers host specificity?
SopD^a^	Outside SPI-1/SPI-2	*Salmonella* SopD2			Promotes membrane fission & macropinosome formation, contributes to *Salmonella* virulence and persistence in mice, induces fluid secretion, promotes invasion of T84 cells
SspH1^a^	Bacteriophage Gifsy-3	*Salmonella* SspH2, SlrP, GogB, *Yersinia* YopM, *Shigella* IpaH_7.8/9.8_	E3 ubiquitin ligase	PKN1	Downregulates proinflammatory responses
SteA (STM1583)^a^	Outside SPI-2				Required for efficient mouse spleen colonisation
SteB (STM1629)^a^	Outside SPI-2		Putative picolinate reductase		

a Can also be translocated via the SPI-2-encoded T3SS.

**Table 2:** Effectors requiring the SPI-2-encoded T3SS for their translocation.

Effector	Gene location	Selected homologues	Activity	Host cell target(s)	Role in infection
GogB	Bacteriophage Gifsy-1	N-terminus: *Yersinia* YopM, *Shigella* IpaH_7.8/9.8_, *Salmonella* SspH1/2, SlrP. C-terminus: *Yersinia* YP2634/Y1471, rabbit EPEC OrfL, EHEC 0157:H7 Z1829			
PipB	SPI-5	*Salmonella* PipB2			
PipB2	Outside SPI-2	*Salmonella* PipB		Kinesin-1	Promotes Sif extension, recruits kinesin-1 to SCV
SifA	Outside SPI-2	*Salmonella* SifB	Rab mimic?	SKIP, Rab7/9	Required for SCV membrane integrity & Sif formation, maintains perinuclear SCV positioning, redirects exocytic vesicles to SCV
SifB	Outside SPI-2	*Salmonella* SifA			
SopD2	Outside SPI-2	*Salmonella* SopD			Contributes to Sif formation, required for efficient bacterial replication in macrophages & mice
SpiC (SsaB)	SPI-2			Hook 3, TassC	Interferes with vesicular trafficking, role in SCV-associated actin polymerisation (VAP) and Sif formation, controls order of protein export through SPI-2 T3SS
SseF	SPI-2				Contributes to Sif formation, recruits dynein to SCV, maintains perinuclear SCV positioning, required for formation of microtubule bundles around SCV, redirects exocytic transport vesicles to SCV
SseG	SPI-2				As SseF
SseI (SrfH/GtgB)	Bacteriophage Gifsy-2			Filamin, TRIP6	Remodels SCV associated F-actin? Promotes phagocyte motility
SseJ	Outside SPI-2		Deacylase, phospholipase A & glycerol-phospholipid :cholesterol acyltransferase	Cholesterol	Negative regulation of Sifs, antagonises SifA SCV stabilisation
SseK1	Outside SPI-2	*Salmonella* SseK2/3, *Citrobacter rodentium* NleB, EHEC Z4328			
SseK2	Outside SPI-2	*Salmonella* SseK1/3, *Citrobacter rodentium* NleB, EHEC Z4328			
SseK3 (NleB)^a^	ST64B coliform bacteriophage	*Salmonella* SseK1/2, *Citrobacter rodentium* NleB, EHEC Z4328			
SseL	Outside SPI-2		Cysteine protease with deubiquitinase activity	IκBα	Macrophage apoptosis, downregulates inflammatory responses
SspH2	In vicinity of bacteriophage remnants	*Salmonella* SspH1, SlrP, GogB, *Yersinia* YopM, *Shigella* IpaH_7.8/9.8_	Inhibits actin polymerisation *in vitro*	Filamin, Profilin	Remodels SCV associated F actin?
SteC (STM1698)	Outside SPI-2	Eukaryotic kinases	Serine/Threonine kinase		Required for VAP
SpvB^b^	pSLT (*S. typhimurium*)	N-terminus: *Photorhabdus luminescens* TcaC. N-terminus: *Bacillus cereus* VipB2, *Clostridium botulinum* C2 toxin component 1/C3 toxin, *Clostridium perfringens* Iota	ADP ribosyl transferase, inhibits actin polymerisation *in vitro*, depolymerises F-actin upon transfection	Actin	Inhibition of VAP, apoptosis of infected cells, required for full virulence in mice
SpvC^b^	pSLT *(S. typhimurium*)	*Shigella* OspF, *Pseudomonas syringae* HopAI1	Phosphothreonine lyase		Required for full virulence in mice

a Found in *S. typhimurium* SL1344, not LT2.

b Found in non-typhoid *Salmonella* serovars.
