# Supplementary material for: Salmonella takes control: effector-driven manipulation of the host
Source: Curr Opin Microbiol. 2009 Feb;12(1):117–24. doi: 10.1016/j.mib.2008.12.001 (PMC2647982; doi:10.1016/j.mib.2008.12.001)
Supplement: Supplementary file 1 [file mmc1.doc]

Supplementary Table 1: Effectors requiring the SPI-1-encoded T3SS for their translocation

| **Effector** | Gene location | | **Selected**  **Homologues** | **Activity** | **Host cell target(s)** | | **Role in infection** | **Refs.** |
| --- | --- | --- | --- | --- | --- | --- | --- | --- |
| AvrA | SPI-1 | | *Yersinia* YopJ/P | Cysteine protease with deubiquitinase activity, acetyltransferase | MKK4/7  IκBα β-catenin | | Inhibits inflammation, represses apoptosis & epithelial innate immunity, stabilises tight junctions | 35, 41•, 45 |
| SipA (SspA) | SPI-1 | | *Shigella* IpaA | Actin binding / stabilising | Actin,  T-plastin | | Increases internalisation efficiency, enhances actin assembly, potentiates SipC activity, triggers PMN transmigration, maintains perinuclear SCV positioning, disrupts tight junctions | 3-7, 23, 33, 34, 47-60 |
| SipB (SspB) | SPI-1 | | *Shigella* IpaB | SPI-1 TTSS translocon component | Cholesterol | | SPI-1 effector delivery, apoptosis of phagocytes | 4, 36, 61-64 |
| SipC (SspC) | SPI-1 | | *Shigella* IpaC | SPI-1 TTSS translocon component, actin nucleation & bundling | Actin | | SPI-1 effector delivery, induces membrane ruffling | 3, 49, 50, 53  64, 65 |
| SipD (SspD) | SPI-1 | | *Shigella* IpaD |  |  | | Regulates SPI-1 effector secretion | 64 |
| SopA | Outside SPI-1 | | Putative EHEC effector (Genbank NP_309587.1) | E3 ubiquitin ligase | HsRMA1 | | Disrupts SCV integrity, induces PMN transmigration | 4, 21•, 48, 56, 66 |
| SopB (SigD) | SPI-5 | | *Shigella* spp. IpgD, cellular 4-phosphatases, synaptojanin | Inositol polyphosphate phosphatase | Inositol phosphates | | Promotes membrane fission & macropinosome formation, maintains perinuclear SCV positioning, promotes epithelial cell survival, triggers nitric oxide production in macrophages, promotes fluid secretion, disrupts tight junctions | 4, 6, 8•, 9, 14••, 15, 16, 17••, 30•, 34, 48-50, 56, 67-73 |
| SopE | Bacteriophage SopEφ | | *Salmonella* SopE2 | Guanine exchange factor (GEF) mimic | Rac-1, Cdc42 | | Induces membrane ruffling & proinflammatory responses, promotes fusion of SCV with early endosomes, disrupts tight junctions | 6, 8•, 13, 34, 47, 49, 50, 68, 74, 75 |
| SopE2 | In vicinity of bacteriophage remnants | | *Salmonella* SopE | GEF mimic | Cdc42 | | Induces membrane ruffling & proinflammatory responses, increases macrophage iNos expression, disrupts tight junctions | 4, 6, 8•, 34, 48, 49, 56,  76, 77 |
| SptP | SPI-1 | | N-terminus: *Yersinia* YopE, *Pseudomonas aeruginosa* ExoS. C-terminus: cellular tyrosine phosphatases, *Yersinia* YopH | GTPase activating protein (GAP) mimic, tyrosine phosphatase | Cdc42, Rac-1, vimentin | | Returns host cytoskeleton to resting state following bacterial entry, downregulates proinflammatory responses | 6, 39, 40, 47, 49, 50,  75, 78 |
| SlrP* |  | *Salmonella* SspH1/H2, GogB*, Yersinia* YopM, *Shigella* IpaH_7.8/9.8_, | | Ubiquitin ligase? |  | Confers host specificity? | | 24, 25 |
| SopD* | Outside  SPI-1/SPI-2 | *Salmonella* SopD2 | |  |  | Promotes membrane fission & macropinosome formation, contributes to *Salmonella* virulence and persistence in mice, induces fluid secretion, promotes invasion of T84 cells | | 4, 10, 32, 48, 56 |
| SspH1* | Bacteriophage Gifsy-3 | *Salmonella* SspH2, SlrP, GogB*, Yersinia* YopM, *Shigella* IpaH_7.8_/_9.8_ | | E3 ubiquitin ligase | PKN1 | Downregulates proinflammatory responses | | 24, 46, 79 |
| SteA* (STM1583) | Outside SPI-2 |  | |  |  | Required for efficient mouse spleen colonisation | | 80 |
| SteB*  (STM1629) | Outside SPI-2 |  | | Putative picolinate reductase |  |  | | 80 |

* Can also be translocated via the SPI-2-encoded T3SS

Supplementary Table 2: Effectors requiring the SPI-2-encoded T3SS for their translocation

| **Effector** | Gene location | **Selected**  **Homologues** | **Activity** | **Host cell target(s)** | **Role in infection** | **Refs.** |
| --- | --- | --- | --- | --- | --- | --- |
| GogB | Bacteriophage Gifsy-1 | N-terminus: *Yersinia* YopM, *Shigella* IpaH_7.8_/_9.8_, *Salmonella* SspH1/2, SlrP. C-terminus: *Yersinia* YP2634/Y1471, rabbit EPEC OrfL, EHEC 0157:H7 Z1829 |  |  |  | 25, 81 |
| PipB | SPI-5 | *Salmonella* PipB2 |  |  |  | 11, 31, 82 |
| PipB2 | Outside SPI-2 | *Salmonella* PipB |  | Kinesin-1 | Promotes Sif extension, recruits kinesin-1 to SCV | 28•, 31, 82 |
| SifA | Outside SPI-2 | *Salmonella* SifB | Rab mimic? | SKIP, Rab7/9 | Required for SCV membrane integrity & Sif formation, maintains perinuclear SCV positioning, redirects exocytic vesicles to SCV | 11, 18, 23-25, 26•, 28•, 29••, 83-87 |
| SifB | Outside SPI-2 | *Salmonella* SifA |  |  |  | 24, 88 |
| SopD2 | Outside SPI-2 | *Salmonella* SopD |  |  | Contributes to Sif formation, required for efficient bacterial replication in macrophages & mice | 32 |
| SpiC (SsaB) | SPI-2 |  |  | Hook 3, TassC | Interferes with vesicular trafficking, role in SCV-associated actin polymerisation (VAP) and Sif formation, controls order of protein export through SPI-2 TTSS | 11, 89-91 |
| SseF | SPI-2 |  |  |  | Contributes to Sif formation, recuits dynein to SCV, maintains perinuclear SCV positioning, required for formation of microtubule bundles around SCV, redirects exocytic transport vesicles to SCV | 25, 26•, 27, 92-94 |
| SseG | SPI-2 |  |  |  | As SseF | 25, 26•, 27, 92, 94, 95 |
| SseI (SrfH/ GtgB) | Bacteriophage Gifsy-2 |  |  | Filamin, TRIP6 | Remodels SCV associated F-actin? Promotes phagocyte motility | 24 |
| SseJ |  |  | Deacylase, phospholipase A & glycerol-phospholipid :cholesterol acyltransferase | Cholesterol | Negative regulation of Sifs, antagonises SifA SCV stabilisation | 18-20, 25, 88, 96, 97 |
| SseK1 | Outside SPI-2 | *Salmonella* SseK2/3, *Citrobacter rodentium* NleB, EHEC Z4328 |  |  |  | 98 |
| SseK2 | Outside SPI-2 | *Salmonella* SseK1/3, *Citrobacter rodentium* NleB, EHEC Z4328 |  |  |  | 98 |
| SseK3* (NleB) | ST64B coliform bacteriophage | *Salmonella* SseK1/2, *Citrobacter rodentium* NleB, EHEC Z4328 |  |  |  | 98 |
| SseL | Outside SPI-2 |  | Cysteine protease with deubiquitinase activity | IκBα | Macrophage apoptosis, downregulates inflammatory responses | 38•, 44 |
| SspH2 | In vicinity of bacteriophage remnants | *Salmonella* SspH1, SlrP, GogB*, Yersinia* YopM, *Shigella* IpaH_7.8_/_9.8_, | Inhibits actin polymerisation *in vitro* | Filamin, Profilin | Remodels SCV associated F-actin? | 24 |
| SteC  (STM1698) | Outside SPI-2 | Eukaryotic kinases | Serine/Threonine kinase |  | Required for VAP | 22, 80 |
| SpvB^#^ | pSLT (*S.typhimurium*) | N-terminus: *Photorhabdus luminescens* TcaC. N-terminus: *Bacillus cereus* VipB2, *Clostridium botulinum* C2 toxin component 1/C3 toxin, *Clostridium perfringens* Iota | ADP ribosyl transferase, inhibits actin polymerisation *in vitro*, depolymerises F-actin upon transfection | Actin | Inhibition of VAP, apoptosis of infected cells, required for full virulence in mice | 24, 25, 37, 96, 99 |
| SpvC^#^ | pSLT (*S.typhimurium*) | *Shigella* OspF, *Pseudomonas syringae* HopAI1 | Phosphothreonine lyase |  | Required for full virulence in mice | 42, 43, 100 |

* Found in *S.typhimurium* SL1344, not LT2

^#^ Found in non-typhoid *Salmonella* serovars

References and recommended reading

Papers of particular interest have been highlighted as:

• of special interest

•• of outstanding interest

1. Filloux A, Hachani A, Bleves S: **The bacterial type VI secretion machine: yet another player for protein transport across membranes.** *Microbiology* 2008, 154:1570-1583.
2. Gal-Mor O, Gibson DL, Baluta D, Vallance BA, Finlay BB: **A novel secretion pathway of *Salmonella enterica* acts as an antivirulence modulator during salmonellosis.** *PLoS Pathog* 2008, 4:e1000036.
3. Hayward RD, Koronakis V: **Direct modulation of the host cell cytoskeleton by *Salmonella* actin-binding proteins.** *Trends Cell Biol* 2002, 12:15-20.
4. Layton AN, Galyov EE: ***Salmonella*-induced enteritis: molecular pathogenesis and therapeutic implications.** *Expert Rev Mol Med* 2007, 9:1-17.
5. McGhie EJ, Hayward RD, Koronakis V: **Control of actin turnover by a *Salmonella* invasion protein.** *Mol Cell* 2004, 13:497-510.
6. Patel JC, Galán JE: **Manipulation of the host actin cytoskeleton by *Salmonella* - all in the name of entry.** *Curr Opin Microbiol* 2005, 8:10-15.
7. Popp D, Yamamoto A, Iwasa M, Nitanai Y, Maéda Y: **Single molecule polymerization, annealing and bundling dynamics of SipA induced actin filaments.** *Cell Motil Cytoskeleton* 2008, 65:165-177.

•8. Patel JC, Galán JE: **Differential activation and function of Rho GTPases during *Salmonella*-host cell interactions.** *J Cell Biol* 2006, 175:453-463.

Using RNAi, the authors demonstrate that Rac and RhoG activation, by SopE and SopB respectively, contributes towards the cytoskeletal remodelling that occurs during *Salmonella* entry. In contrast, Cdc42 upregulation is required for eliciting pro-inflammatory nuclear responses associated with *Salmonella* infection.

1. Dai S, Zhang Y, Weimbs T, Yaffe MB, Zhou D: **Bacteria-generated PtdIns(3)P recruits VAMP8 to facilitate phagocytosis.** *Traffic* 2007, 8:1365-1374.
2. Bakowski MA, Cirulis JT, Brown NF, Finlay BB, Brumell JH: **SopD acts cooperatively with SopB during *Salmonella enterica* serovar Typhimurium invasion.** *Cell Microbiol* 2007, 9:2839-2855.
3. Steele-Mortimer O: **The *Salmonella*-containing vacuole: moving with the times.** *Curr Opin Microbiol* 2008, 11:38-45.

•12. Drecktrah D, Knodler LA, Howe D, Steele-Mortimer O: ***Salmonella* trafficking is defined by continuous dynamic interactions with the endolysosomal system.** *Traffic* 2007, 8:212-225.

This study uses high-resolution live cell imaging to show that the SCV undergoes continuous interaction with late endosomes/lysosomes following *Salmonella* invasion.

1. Mukherjee K, Parashuraman S, Raje M, Mukhopadhyay A: **SopE acts as an Rab5-specific nucleotide exchange factor and recruits non-prenylated Rab5 on *Salmonella*-containing phagosomes to promote fusion with early endosomes.** *J Biol Chem* 2001, 276: 23607-23615.

••14. Mallo GV, Espina M, Smith AC, Terebiznik MR, Alemán A, Finlay BB, Rameh LE, Grinstein S, Brumell JH: **SopB promotes phosphatidylinositol 3-phosphate formation on *Salmonella* vacuoles by recruiting Rab5 and Vps34.** *J Cell Biol* 2008, 182:741-752.

In this study the authors show that the SPI-1 effector SopB is necessary for PI(3)P accumulation on the SCV membrane, a critical requirement for vacuolar maturation. SopB inositol phosphatase activity recruits Rab5 to the SCV. Rab5 in turn associates with the PI3-kinase Vps34, which is responsible for PI(3)P production on the SCV membrane.

1. Dukes JD, Lee H, Hagen R, Reaves BJ, Layton AN, Galyov EE, Whitley P: **The secreted *Salmonella dublin* phosphoinositide phosphatase, SopB, localizes to PtdIns(3)P-containing endosomes and perturbs normal endosome to lysosome trafficking.** *Biochem J* 2006, 395:239-247.
2. Bujny MV, Ewels PA, Humphrey S, Attar N, Jepson MA, Cullen PJ: **Sorting nexin-1 defines an early phase of *Salmonella*-containing vacuole-remodeling during *Salmonella* infection**. *J Cell Sci* 2008, 121:2027-2036.

••17. Kuijl C, Savage ND, Marsman M, Tuin AW, Janssen L, Egan DA, Ketema M, van den Nieuwendijk R, van den Eeden SJ, Geluk A *et al*: **Intracellular bacterial growth is controlled by a kinase network around PKB/AKT1**. *Nature* 2007, 450:725-730.

Using an RNAi screen, this elegant study identified that the host kinase AKT1/PKB plays a central role in controlling intracellular bacterial growth. SopB was shown to activate AKT1, which subsequently delays phagosomal-lysosomal fusion through the AS160-RAB14 pathway.

1. Ruiz-Albert J, Yu XJ, Beuzón CR, Blakey AN, Galyov EE, Holden DW: **Complementary activities of SseJ and SifA regulate dynamics of the *Salmonella typhimurium* vacuolar membrane.** *Mol Microbiol* 2002, 44:645-661.
2. Ohlson MB, Fluhr K, Birmingham CL, Brumell JH, Miller SI: **SseJ deacylase activity by *Salmonella enterica* serovar Typhimurium promotes virulence in mice.** *Infect Immun* 2005, 73:6249-6259.
3. Lossi NS, Rolhion N, Magee AI, Boyle C, Holden DW: **The *Salmonella* SPI-2 effector SseJ exhibits eukaryotic activator-dependent phospholipase A and glycerophospholipid : cholesterol acyltransferase activity.** *Microbiology* 2008, 154:2680-2688.

•21. Diao J, Zhang Y, Huibregtse JM, Zhou D, Chen J: **Crystal structure of SopA, a *Salmonella* effector protein mimicking a eukaryotic ubiquitin ligase.** *Nature Struct Mol Biol* 2008, 15:65-70.

In this study, the authors determine the crystal structure of the SPI-1 effector SopA and show that it has a high structural similarity to eukaryotic HECT E3 ligases. SopA therefore is an elegant example of a bacterial protein that has evolved as a structural and functional mimic of a eukaryotic enzyme.

1. Poh J, Odendall C, Spanos A, Boyle C, Liu M, Freemont P, Holden DW: **SteC is a *Salmonella* kinase required for SPI-2-dependent F-actin remodelling.** *Cell Microbiol* 2008, 10:20-30.
2. Brawn LC, Hayward RD, Koronakis V: ***Salmonella* SPI1 effector SipA persists after entry and cooperates with a SPI2 effector to regulate phagosome maturation and intracellular replication.** *Cell Host Microbe* 2007, 1:63-75.
3. Miao EA, Brittnacher M, Haraga A, Jeng RL, Welch MD, Miller SI: ***Salmonella* effectors translocated across the vacuolar membrane interact with the actin cytoskeleton**. *Mol Microbiol* 2003, 48:401-415.
4. Ramsden AE, Holden DW, Mota LJ: **Membrane dynamics and spatial distribution of *Salmonella*-containing vacuoles.** *Trends Microbiol* 2007, 15:516-524.

•26. Kuhle V, Abrahams GL, Hensel M: **Intracellular *Salmonella* *enterica* redirect exocytic transport processes in a *Salmonella* pathogenicity island 2-dependent manner.** *Traffic* 2006, 7:716-730.

In this paper, the authors show that exocytic transport vesicles are redirected to the SCV through the combined actions of the SPI-2 effectors SifA, SseF and SseG.

1. Deiwick J, Salcedo SP, Boucrot E, Gilliland SM, Henry T, Petermann N, Waterman SR. Gorvel JP, Holden DW, Méresse S: **The translocated *Salmonella* effector proteins SseF and SseG interact and are required to establish an intracellular replication niche.** *Infect Immun* 2006, 74:6965-6972.

•28. Henry T, Couillault C, Rockenfeller P, Boucrot E, Dumont A, Schroeder N, Hermant A, Knodler LA, Lecine P, Steele-Mortimer O *et al*: **The *Salmonella* effector protein PipB2 is a linker for kinesin-1.** *Proc Natl Acad Sci USA* 2006, 103:13497-13502.

These authors show that the SPI-2 effector PipB2 is required for the direct recruitment of kinesin-1 to the SCV, and therefore behaves as a SifA antagonist.

••29. Boucrot E, Henry T, Borg JP, Gorvel JP, Méresse S: **The intracellular fate of *Salmonella* depends on the recruitment of kinesin.** *Science* 2005, 308:1174-1178.

In this paper, the authors convincingly show that the SPI-2 effector SifA interacts with a host protein of previously unknown function, which they subsequently named SKIP (SifA and kinesin–interacting protein). SKIP was demonstrated to downregulate kinesin recruitment to the SCV, ensuring maintenance of vacuolar integrity and perinuclear SCV positioning.

•30. Wasylnka JA, Bakowski MA, Szeto J, Ohlson MB, Trimble WS, Miller SI, Brumell JH: **Role for myosin II in regulating positioning of *Salmonella*-containing vacuoles and intracellular replication.** *Infect Immun* 2008, 76:2722-2735.

The authors demonstrate that the actin-based motor protein myosin II is recruited to the SCV and contributes towards the maintenance of SCV positioning and membrane integrity by counteracting the activity of the SPI-2 effectors PipB2 and SseJ. SopB was found to be required for SCV positioning and was sufficient to activate myosin II via phosphorylation of its regulatory light chain (MLC), most likely via the Rho/ROCK/MLC signalling pathway.

1. Knodler LA, Steele-Mortimer O: **The *Salmonella* effector PipB2 affects late endosome/lysosome distribution to mediate Sif extension.** *Mol Biol Cell* 2005, 16:4108-4123.
2. Jiang X, Rossanese OW, Brown NF, Kujat-Choy S, Galán JE, Finlay BB, Brumell JH: **The related effector proteins SopD and SopD2 from *Salmonella* *enterica* serovar Typhimurium contribute to virulence during systemic infection of mice.** *Mol Microbiol* 2004, 54:1186-1198.
3. Wall DM, Nadeau WJ, Pazos MA, Shi HN, Galyov EE, McCormick BA: **Identification of the *Salmonella enterica* serotype Typhimurium SipA domain responsible for inducing neutrophil recruitment across the intestinal epithelium.** *Cell Microbiol* 2007, 9:2299-2313.
4. Boyle EC, Brown NF, Finlay BB: ***Salmonella enterica* serovar Typhimurium effectors SopB, SopE, SopE2 and SipA disrupt tight junction structure and function.** *Cell Microbiol* 2006, 8:1946-1957.
5. Liao AP, Petrof EO, Kuppireddi S, Zhao Y, Xia Y, Claud EC, Sun J: ***Salmonella* type III effector AvrA stabilizes cell tight junctions to inhibit inflammation in intestinal epithelial cells.** *PLoS ONE* 2008, 3:e2369.
6. Fink SL, Cookson BT: **Pyroptosis and host cell death responses during *Salmonella* infection.** *Cell Microbiol* 2007, 9:2562-2570.
7. Browne SH, Hasegawa P, Okamoto S, Fierer J, Guiney DG: **Identification of *Salmonella* SPI-2 secretion system components required for SpvB-mediated cytotoxicity in macrophages and virulence in mice.** *FEMS Immunol Med Microbiol* 2008, 52:194-201.

•38. Rytkönen A, Poh J, Garmendia J, Boyle C, Thompson A, Liu M, Freemont P, Hinton JC, Holden DW: **SseL, a *Salmonella* deubiquitinase required for macrophage killing and virulence.** *Proc Natl Acad Sci USA* 2007,104:3502-3507.

The identification of a previously uncharacterised SPI-2 effector, SseL, is described in this paper. SseL exhibits sequence similarities to cysteine proteases with deubiquitinating activity and was shown to possess deubiquitinase activity both *in vitro* and *in vivo*. In addition, SseL induced delayed cytotoxicity in macrophages and *Salmonella* strains lacking *sseL* were attenuated for virulence in mice.

1. Murli S, Watson RO, Galán JE: **Role of tyrosine kinases and the tyrosine phosphatase SptP in the interaction of *Salmonella* with host cells.** *Cell Microbiol* 2001, 3:795-810.
2. Lin SL, Le TX, Cowen DS: **SptP, a *Salmonella typhimurium* type III-secreted protein, inhibits the mitogen-activated protein kinase pathway by inhibiting Raf activation.** *Cell Microbiol* 2003, 5:267-275.

•41. Jones RM, Wu H, Wentworth C, Luo L, Collier-Hyams L, Neish AS: ***Salmonella* AvrA Coordinates Suppression of Host Immune and Apoptotic Defenses via JNK Pathway Blockade.** *Cell Host Microbe* 2008, 3:233-244.

These authors show that the SPI-1 effector AvrA possesses acetyltransferase activity toward specific mitogen-activated protein kinase kinases (MAPKKs) and potently inhibits c-Jun N-terminal kinase (JNK) and NFκB signalling pathways in order to downregulate apoptosis of *Salmonella*-infected cells.

1. Mazurkiewicz P, Thomas J, Thompson JA, Liu M, Arbibe L, Sansonetti P, Holden DW: **SpvC is a *Salmonella* effector with phosphothreonine lyase activity on host mitogen-activated protein kinases.** *Mol Microbiol* 2008, 67:1371-1383.
2. Li H, Xu H, Zhou Y, Zhang J, Long C, Li S, Chen S, Zhou JM, Shao F: **The phosphothreonine lyase activity of a bacterial type III effector family.** *Science* 2007, 315:1000-1003.
3. Le Negrate G, Faustin B, Welsh K, Loeffler M, Krajewska M, Hasegawa P, Mukherjee S, Orth K, Krajewski S, Godzik A, *et al*: ***Salmonella* secreted factor L deubiquitinase of *Salmonella typhimurium* inhibits NF-κB, suppresses IκBα ubiquitination and modulates innate immune responses.** *J Immunol* 2008, 180:5045-5056.
4. Ye Z, Petrof EO, Boone D, Claud EC, Sun J: ***Salmonella* effector AvrA regulation of colonic epithelial cell inflammation by deubiquitination.** *Am J Pathol* 2007, 171: 882-892.
5. Rohde JR, Breitkreutz A, Chenal A, Sansonetti PJ, Parsot C: **Type III secretion effectors of the IpaH family are E3 ubiquitin ligases.** *Cell Host Microbe* 2007, 1:77-83.
6. Winnen B, Schlumberger MC, Sturm A, Schüpbach K, Siebenmann S, Jenny P, Hardt WD: **Hierarchical effector protein transport by the *Salmonella* Typhimurium SPI-1 Type III secretion system.** *PLoS ONE* 2008, 3:e2178.
7. Raffatellu M, Wilson RP, Chessa D, Andrews-Polymenis H, Tran QT, Lawhon S, Khare S, Adams LG, Bäumler AJ: **SipA, SopA, SopB, SopD, and SopE2 contribute to *Salmonella enterica* serotype Typhimurium invasion of epithelial cells.** *Infect Immun* 2005, 73:146-154.
8. Cain RJ, Hayward RD, Koronakis V: **The target cell plasma membrane is a critical interface for *Salmonella* cell entry effector-host interplay.** *Mol Microbiol* 2004, 54:887-904.
9. Cain RJ, Hayward RD, Koronakis V: **Deciphering interplay between *Salmonella* invasion effectors.** *PLoS Pathog*. 2008, 4:e1000037.
10. Zhou D, Mooseker MS, Galán JE: **Role of the *S. typhimurium* actin-binding protein SipA in bacterial internalization.** *Science* 1999, 283:2092-2095.
11. Hapfelmeier S, Hardt WD: **A mouse model for *S. typhimurium*-induced enterocolitis**. *Trends Microbiol* 2005, 13:497-503.
12. McGhie EJ, Hayward RD, Koronakis V: **Cooperation between actin-binding proteins of invasive *Salmonella*:** **SipA potentiates SipC nucleation and bundling of actin.** *EMBO J* 2001, 20:2131-2139.
13. Lilic M, Galkin VE, Orlova A, VanLoock MS, Egelman EH, Stebbins CE: ***Salmonella* SipA polymerizes actin by stapling filaments with nonglobular protein arms.** *Science* 2003, 301:1918-1921.
14. Zhou D, Mooseker MS, Galán JE: **An invasion-associated *Salmonella* protein modulates the actin-bundling activity of plastin.** *Proc Natl Acad Sci USA* 1999, 96:10176-10181.
15. Zhang S, Santos RL, Tsolis RM, Stender S, Hardt WD, Bäumler AJ, Adams LG: **The *Salmonella enterica* serotype Typhimurium effector proteins SipA, SopA, SopB, SopD, and SopE2 act in concert to induce diarrhea in calves.** *Infect Immun* 2002, 70:3843-3855.
16. Silva M, Song C, Nadeau WJ, Matthews JB, McCormick BA: ***Salmonella typhimurium* SipA-induced neutrophil transepithelial migration: involvement of a PKC-α-dependent signal transduction pathway.** *Am J Physiol Gastrointest Liver Physiol* 2004, 286:G1024-1031.
17. Lee CA, Silva M, Siber AM, Kelly AJ, Galyov E, McCormick BA: **A secreted *Salmonella* protein induces a proinflammatory response in epithelial cells, which promotes neutrophil migration.** *Proc Natl Acad Sci USA.* 2000, 97:12283-12288.
18. Galkin VE, Orlova A, VanLoock MS, Zhou D, Galán JE, Egelman EH: **The bacterial protein SipA polymerizes G-actin and mimics muscle nebulin.** *Nat Struct Biol* 2002, 9:518-521.
19. Schlumberger MC, Käppeli R, Wetter M, Müller AJ, Misselwitz B, Dilling S, Kremer M, Hardt WD: **Two newly identified SipA domains (F1, F2) steer effector protein localization and contribute to *Salmonella* host cell manipulation.** *Mol Microbiol* 2007, 65:741-760.
20. Hersh D, Monack DM, Smith MR, Ghori N, Falkow S, Zychlinsky A: **The *Salmonella* invasin SipB induces macrophage apoptosis by binding to caspase-1.** *Proc Natl Acad Sci USA* 1999, 96:2396-2401.
21. Hernandez LD, Pypaert M, Flavell RA, Galán JE: **A *Salmonella* protein causes macrophage cell death by inducing autophagy.** *J Cell Biol* 2003, 163:1123-1131.
22. Hayward RD, Cain RJ, McGhie EJ, Phillips N, Garner MJ, Koronakis V: **Cholesterol binding by the bacterial type III translocon is essential for virulence effector delivery into mammalian cells.** *Mol Microbiol* 2005, 56:590-603.
23. Mueller CA, Broz P, Cornelis GR: **The type III secretion system tip complex and translocon.** *Mol Microbiol* 2008, 68:1085-1095.
24. Hayward RD, Koronakis V: **Direct nucleation and bundling of actin by the SipC protein of invasive *Salmonella***. *EMBO J* 1999, 18:4926-4934.
25. Zhang Y, Higashide W, Dai S, Sherman DM, Zhou D: **Recognition and ubiquitination of *Salmonella* type III effector SopA by a ubiquitin E3 ligase, HsRMA1.** *J Biol Chem* 2005, 280:38682-38688.
26. Norris FA, Wilson MP, Wallis TS, Galyov EE, Majerus PW: **SopB, a protein required for virulence of *Salmonella dublin*, is an inositol phosphate phosphatase**. *Proc Natl Acad Sci U S A* 1998, 95:14057-14059.
27. Zhou D, Chen LM, Hernandez L, Shears SB, Galán JE: **A *Salmonella* inositol polyphosphatase acts in conjunction with other bacterial effectors to promote host cell actin cytoskeleton rearrangements and bacterial internalization.** *Mol Microbiol* 2001, 39:248-259.
28. Terebiznik MR, Vieira OV, Marcus SL, Slade A, Yip CM, Trimble WS, Meyer T, Finlay BB, Grinstein S: **Elimination of host cell PtdIns(4,5)P(2) by bacterial SigD promotes membrane fission during invasion by *Salmonella*.** *Nature Cell Biol* 2002, 4:766-773.
29. Hernandez LD, Hueffer K, Wenk MR, Galán JE: ***Salmonella* modulates vesicular traffic by altering phosphoinositide metabolism.** *Science* 2004, 304:1805-1807.
30. Steele-Mortimer O, Knodler LA, Marcus SL, Scheid MP, Goh B, Pfeifer CG, Duronio V, Finlay BB: **Activation of Akt/protein kinase B in epithelial cells by the *Salmonella typhimurium* effector SigD**. *J Biol Chem 2000*, 275:37718-37724.
31. Mason D, Mallo GV, Terebiznik MR, Payrastre B, Finlay BB, Brumell JH, Rameh L, Grinstein S: **Alteration of epithelial structure and function associated with PtdIns(4,5)P_2_ degradation by a bacterial phosphatase.** *J Gen Physiol* 2007, 129:267-283.
32. Marcus SL, Wenk MR, Steele-Mortimer O, Finlay BB: **A synaptojanin-homologous region of *Salmonella typhimurium* SigD is essential for inositol phosphatase activity and Akt activation.** *FEBS Lett* 2001, 494:201-207.
33. Hardt WD, Chen LM, Schuebel KE, Bustelo XR, Galán JE: ***S. typhimurium* encodes an activator of Rho GTPases that induces membrane ruffling and nuclear responses in host cells.** *Cell* 1998, 93:815-826.
34. Kubori T, Galán JE: **Temporal regulation of *Salmonella* virulence effector function by proteasome-dependent protein degradation**. *Cell* 2003, 15:333-342.
35. Stender S, Friebel A, Linder S, Rohde M, Mirold S, Hardt WD: **Identification of SopE2 from *Salmonella typhimurium*, a conserved guanine nucleotide exchange factor for Cdc42 of the host cell.** *Mol Microbiol* 2000, 36:1206-1221.
36. Bakshi CS, Singh VP, Wood MW, Jones PW, Wallis TS, Galyov EE: **Identification of SopE2, a *Salmonella* secreted protein which is highly homologous to SopE and involved in bacterial invasion of epithelial cells.** *J Bacteriol* 2000, 182:2341-2344.
37. Fu Y, Galán JE: **A *Salmonella* protein antagonizes Rac-1 and Cdc42 to mediate host-cell recovery after bacterial invasion.** *Nature* 1999, 401:293-297.
38. Haraga A, Miller SI: **A *Salmonella* type III secretion effector interacts with the mammalian serine/threonine protein kinase PKN1.** *Cell Microbiol* 2006, 8:837-846.
39. Geddes K, Worley M, Niemann G, Heffron F: **Identification of new secreted effectors in *Salmonella enterica* serovar Typhimurium.** *Infect Immun* 2005, 73:6260-6271.
40. Coombes BK, Wickham ME, Brown NF, Lemire S, Bossi L, Hsiao WW, Brinkman FS, Finlay BB: **Genetic and molecular analysis of GogB, a phage-encoded type III-secreted substrate in *Salmonella enterica* serovar Typhimurium with autonomous expression from its associated phage.** *J Mol Biol* 2005, 348:817-830.
41. Knodler LA, Vallance BA, Hensel M, Jäckel D, Finlay BB, Steele-Mortimer O: ***Salmonella* type III effectors PipB and PipB2 are targeted to detergent-resistant microdomains on internal host cell membranes.** *Mol Microbiol* 2003, 49:685-704.
42. Harrison RE, Brumell JH, Khandani A, Bucci C, Scott CC, Jiang X, Finlay BB, Grinstein S: ***Salmonella* impairs RILP recruitment to Rab7 during maturation of invasion vacuoles.** *Mol Biol Cell* 2004, 15:3146-3154.
43. Brumell JH, Tang P, Mills SD, Finlay BB: **Characterization of *Salmonella*-induced filaments (Sifs) reveals a delayed interaction between *Salmonella*-containing vacuoles and late endocytic compartments**. *Traffic* 2001, 2:643-653.
44. Knodler LA, Steele-Mortimer O: **The *Salmonella* effector PipB2 affects late endosome/lysosome distribution to mediate Sif extension.** Mol Biol Cell 2005, 16:4108-4123.
45. Stein MA, Leung KY, Zwick M, Garcia-del Portillo F, Finlay BB: **Identification of a *Salmonella* virulence gene required for formation of filamentous structures containing lysosomal membrane glycoproteins within epithelial cells.** *Mol Microbiol* 1996, 20:151-164.
46. Brumell JH, Rosenberger CM, Gotto GT, Marcus SL, Finlay BB: **SifA permits survival and replication of *Salmonella typhimurium* in murine macrophages.** *Cell Microbiol* 2001, 3:75-84.
47. Freeman JA, Ohl ME, Miller SI: **The *Salmonella enterica* serovar Typhimurium translocated effectors SseJ and SifB are targeted to the *Salmonella*-containing vacuole.** *Infect Immun* 2003, 71:418-427.
48. Uchiya K, Barbieri MA, Funato K, Shah AH, Stahl PD, Groisman EA: **A *Salmonella* virulence protein that inhibits cellular trafficking.** *EMBO J* 1999, 18:3924-3933.
49. Lee AH, Zareei MP, Daefler S: **Identification of a NIPSNAP homologue as host cell target for *Salmonella* virulence protein SpiC.** *Cell Microbiol* 2002, 4:739-750.
50. Shotland Y, Krämer H, Groisman EA: **The *Salmonella* SpiC protein targets the mammalian Hook3 protein function to alter cellular trafficking.** *Mol Microbiol* 2003, 49:1565-1576.
51. Kuhle V, Hensel M: **SseF and SseG are translocated effectors of the type III secretion system of *Salmonella* pathogenicity island 2 that modulate aggregation of endosomal compartments.** *Cell Microbiol* 2002, 4:813-824.
52. Abrahams GL, Muller P, Hensel M: **Functional dissection of SseF, a Type III effector protein involved in positioning the *Salmonella*-containing vacuole.** *Traffic* 2006, 7:950-965.
53. Kuhle V, Jackel D, Hensel M: **Effector proteins encoded by *Salmonella* pathogenicity island 2 interfere with the microtubule cytoskeleton after translocation into host cells.** *Traffic* 2004, 5:356-370.
54. Salcedo SP, Holden DW: **SseG, a virulence protein that targets *Salmonella* to the Golgi network.** *EMBO J* 2003, 22:5003-5014.
55. Birmingham CL, Jiang X, Ohlson MB, Miller SI, Brumell JH: ***Salmonella*-induced filament formation is a dynamic phenotype induced by rapidly replicating *Salmonella enterica* serovar Typhimurium in epithelial cells.** *Infect Immun* 2005, 73:1204-1208.
56. Nawabi P, Catron DM, Haldar K: **Esterification of cholesterol by a type III secretion effector during intracellular *Salmonella* infection.** *Mol Microbiol* 2008, 68:173-185.
57. Kujat Choy SL, Boyle EC, Gal-Mor O, Goode DL, Valdez Y, Vallance BA, Finlay BB: **SseK1 and SseK2 are novel translocated proteins of *Salmonella enterica* serovar Typhimurium.** *Infect Immun* 2004, 72:5115-5125.
58. Lesnick ML, Reiner NE, Fierer J, Guiney DG: **The *Salmonella* *spvB* virulence gene encodes an enzyme that ADP-ribosylates actin and destabilizes the cytoskeleton of eukaryotic cells.** *Mol Microbiol* 2001, 39:1464-1470.
59. Chen L, Wang H, Zhang J, Gu L, Huang N, Zhou JM, Chai J: **Structural basis for the catalytic mechanism of phosphothreonine lyase.** *Nat Struct Mol Biol* 2008, 15:101-102.
